# Poly (trimethylene carbonate)/doxycycline hydrochloride films in the treatment of Achilles tendon defect in rats

**DOI:** 10.3389/fbioe.2023.1135248

**Published:** 2023-02-24

**Authors:** Jinchi Zhang, Xiaowei Zhang, Wei Li, Jing Guo, Liqun Yang, Guangqi Yan

**Affiliations:** ^1^ Department of Biomaterials, Shengjing Hospital of China Medical University, Shenyang, China; ^2^ NHC Key Laboratory of Reproductive Health and Medical Genetics (China Medical University), Liaoning Research Institute of Family Planning (The Affiliated Reproductive Hospital of China Medical University), Shenyang, China; ^3^ College of Kinesiology, Shenyang Sport University, Shenyang, China; ^4^ Department of Oral and Maxillofacial Surgery, School of Stomatology, China Medical University, Shenyang, China

**Keywords:** poly (trimethylene carbonate), doxycycline hydrochloride, Achilles tendon defect, regeneration, treatment

## Abstract

**Introduction:** In this study, Poly (trimethylene carbonate)/Doxycycline hydrochloride (PTMC/DH) films were introduced to repair the Achilles tendon defects for the first time.

**Methods:** (PTMC/DH) films with different DH content of 10, 20, and 30% (w/w) were prepared by solvent casting. The *in vitro* and *in vivo* drug release of the prepared PTMC/DH films was investigated.

**Results:** The results of drug release experiments showed that the PTMC/DH films released effective concentrations of doxycycline for more than 7 and 28 days *in vitro* and *in vivo*, respectively. The results of antibacterial activity experiments showed diameters of 25.00 ± 1.00 mm, 29.33 ± 1.15 mm, and 34.67 ± 1.53 mm, respectively, for the inhibition zones produced by the release solutions of PTMC/DH films with 10, 20 and 30% (w/w) DH at 2 h, indicating that the drug-loaded films could inhibit Staphylococcus aureus well. After treatment, the Achilles tendon defects have recovered well, as indicated by the more robust biomechanical properties and the lower fibroblast density of the repaired Achilles tendons. Pathology revealed that the pro-inflammatory cytokine, IL-1β, and the anti-inflammatory factor, TGF-β1, peaked in the first three days and gradually decreased as the drug was released more slowly.

**Discussion:** These results demonstrated that the PTMC/DH films have great potential for regenerating Achilles tendon defects.

## 1 Introduction

The Achilles tendon, one of the thickest and most powerful tendons in the body, is anatomically located approximately 15 cm above the heel tuberosity and consists mainly of a fusion of the tendons of the deep flounder and superficial gastrocnemius muscles. The incidence of traumatic injury and rupture of the Achilles tendon is increasing, whether or not it is associated with a surrounding soft tissue defect. This problem is challenging for tissue engineering technology and regenerative medicine (Bottagisio and Lovati, 2017). Achilles tendon defects are common in sports and other rigorous movements and can lead to functional impairment and months or years of disability (Dams et al., 2019). In addition to direct injuries, it is estimated that approximately 70% or more of Achilles tendon defects are more commonly involved during sports, especially explosive sports such as ball and track and field (Ganestam et al., 2016). Achilles tendon defects are the most common of all sports injuries, with post-operative Achilles tendon re-rupture rates ranging from approximately 1.7%–5.6% (Cerrato and Switaj, 2017). The injury rate (IR) for Achilles tendon defects in 255 NCAA competitive athletes was calculated to be 2.17% (Chan et al., 2020). It has been calculated that 850 (0.119%) of the 713,456 individuals in the Korean Health Insurance Service-National Sample Cohort database underwent repair of Achilles tendon defects, with the total number of procedures per year increasing with age, peaking and subsequently decreasing at 30–39 years of age (Park et al., 2022). The flexible use of tissue engineering in rehabilitation medicine provides the most favorable support and assurance for repairing Achilles tendon defects. Although there are many surgical approaches to repairing Achilles tendon defects in modern regenerative medicine, there is still no consensus on the simplest, most effective, and safest technique (Chugaev et al., 2018; Monnerie et al., 2019). At this stage, ultrasound imaging is used clinically to examine the early healing of Achilles tendon defects. A unique Achilles tendon defect repair score has also been developed, which assesses the presence or absence of echogenicity in the tendon defect area and the distribution of peritendinous tissue (Hiramatsu et al., 2018). One data showed that in 808 patients with Achilles tendon defects, ultrasound had a sensitivity of 94.8% and a specificity of 98.7% for detecting complete Achilles tendon defects, and the study illustrated the accuracy of ultrasound as a means of providing a diagnostic tool for treating patients with Achilles tendon defects (Aminlari et al., 2021).

Doxycycline hydrochloride (DH) is a commonly used antibiotic in clinical practice. It belongs to the tetracycline group of antibiotics. It is a relatively broad-spectrum antibiotic with a strong antibacterial effect, showing some antibacterial activity against both gram-positive and negative bacteria. Matrix metalloproteinases (MMP) are enzymes involved in the degradation and remodeling of the extracellular matrix (ECM) and play an essential role in the repair of Achilles tendon defects (Molloy et al., 2006; Garofalo et al., 2011; Thomopoulos et al., 2015; Gong et al., 2018; Fernandes de Jesus et al., 2019). Drugs containing MMP have been the first choice in treating Achilles tendinopathy (Smith and Leyden, 2005; Kessler et al., 2014; Nguyen et al., 2017; Baldwin et al., 2019). It is well established in the literature that DH, a drug that inhibits MMP expression *in vivo*, thereby inhibiting the continued expression of inflammatory factors, can have an anti-inflammatory and analgesic effect and reduce swelling. Bedi et al. (2010) (Smith and Leyden, 2005) reported that the immediate administration of doxycycline after surgery could inhibit collagenase activity and play an essential role in improving the structural healing rate. Pasternak et al. (2006) (Bramono et al., 2004) evaluated the effect of doxycycline on Achilles tendon healing in a rat Achilles tendon transection model. This study showed the pharmacological effects of MMP inhibitors on tendon repair for the first time. Sobhani-Eraghi et al. (2020) randomized 20 healthy rats of the same sex into sham-operated and doxycycline groups. The rats underwent surgical intervention with a 2-mm incision on the lateral aspect of the right Achilles tendon. The group was treated with doxycycline 50 mg/kg/d by gavage for 30 days. The study showed significant changes in the repaired tissue in the treated group compared to the sham-operated group, as indicated by a more regular arrangement of collagen fibers, increased cell density, and fibroblast activity. The results indicated that doxycycline improves the extent of the Achilles tendon defect, especially the integrity of the collagen fiber arrangement.

Biodegradable aliphatic polycarbonates have good biodegradability, excellent biocompatibility, and physical properties as an essential class of biodegradable medical polymeric materials (Zhu et al., 1991; Pego et al., 2003; Sachlos and Czernuszka, 2003). Compared with polyesters, such as PLA and PLGA (Albertsson and Eklund, 1995; Athanasiou et al., 1996; Karp et al., 2003), the most significant advantage of biodegradable aliphatic polycarbonates is that no acidic degradation products are generated during the degradation process (Chen et al., 2022), which does not cause side effects such as sterile inflammation in the organism. The most common and widely studied biodegradable aliphatic polycarbonate as a biomedical material is poly (trimethylene carbonate) (PTMC). PTMC has good biocompatibility and biodegradability and is flexible at body temperature. It can be widely used in biodegradable ligature devices, controlled drug release materials, and *in vivo* implant materials. Shieh et al. (1990) found that random copolymers of 90% dimethyltrimethylene carbonate (DMTMC) and 10% trimethylene carbonate (TMC) could be prepared as polymeric material fibers. They retain excellent biomechanical properties and are suitably biodegradable. The basis was laid for the proliferation and migration of peripheral cells in rabbit Achilles tendon defects, further enhancing the activity of stromal cells. Li et al. (2020) prepared a nanofibrous polycaprolactone/poly (trimethyl carbonate) methacrylate (PCL/PTMC-MA) biomaterial polymer scaffold and observed the fibrous morphology of the composite scaffold by scanning electron microscopy. The results showed that this polymer composite scaffold had substantially increased mechanical properties, prevented tissue adhesions, and promoted cell proliferation and differentiation around the Achilles tendon defect. Hence, the application of PTMC as a carrier material for the repair of Achilles tendon defects could be an up-and-coming area of research in the future.

In this study, degradable Doxycycline hydrochloride (DH) loaded poly (trimethylene carbonate) (PTMC) films were developed to achieve slow drug release for a prolonged period. The effect of the DH-loaded PTMC films on the healing process of Achilles tendon rupture after surgical repairment was assessed, confirming that biodegradable PTMC/DH films would enhance the healing process and improve the biomechanical properties of surgically repaired Achilles tendons.

## 2 Materials and methods

### 2.1 Materials

Trimethylene carbonate (TMC) was purchased from Daigang Biomaterials Co., Ltd. (Jinan, Shandong, China), recrystallized twice with ethyl acetate and dried under vacuumed at 37°C for 24 h before copolymerization. The catalyst, stannous iso octanoate Sn(Oct)_2_ (95%) was purchased from Sigma-Aldrich and used as received. Doxycycline hydrochloride (DH) drug was purchased from Aladdin (molecular formula C_22_H_24_N_2_O_8_·HCl·0.5H_2_O·0.5C_2_H_6_O, molecular weight 512.94, purity ≥ 98%) and arrived cold chain and used. All other solvents and reagents were of analytical grade and purified using standard methods.

### 2.2 Preparation of PTMC polymer

The PTMC polymer (molecular weight Mw ≈ 32,000 Da) was synthesized by ring-opening polymerization (Yang et al., 2015). Briefly, TMC (20.4 g, 0.2 mol) and Sn(Oct)_2_ (0.2 M; 1/20,000 eq, 25 μL) were added to a glass ampoule. The ampoule was then heat sealed under a high vacuum (5 mmHg) and immersed in an oil bath at 130°C ± 2°C for 24 h. The oil bath was removed, and the ampoule was cooled to room temperature. The polymer was obtained by crushing the ampoule. Subsequently, the crude polymer product was dissolved in chloroform, further purified in ice-methanol, and dried under vacuum to a constant weight. Mn and PDI were calculated using polystyrene as a standard.

### 2.3 Preparation and characterization of PTMC/DH films

Certain PTMC and 10%, 20%, 30% (w/w) DH were weighted and dissolved in Dichloromethane (DCM). A clear liquid is obtained by magnetic stirring and then left to remove the air bubbles. The solution was poured into a flat-bottomed PTFE Petri dish, allowing the solution to level off naturally, and then placed in a drying oven for 6 h. The drug-carrying film was prepared with an area of 10 mm × 50 mm and a thickness of approximately 0.2 mm. The film was dried under vacuum at room temperature to remove residual organic solvent and water to a constant weight, sealed, and packed at −20°C for storage.

A scanning electron microscope (TESCAN MIRA LMS, Czech Republic) was used to examine the macroscopic morphology of the PTMC and PTMC/DH drug-carrying films. FTIR spectroscopy was performed using a model US Thermo Scientific Nicolet iS50. The resolution in the ATR mode was 4 cm^−1^_,_ and a total of 27 scans were performed. The drug-carrying film samples were compressed into potassium bromide disks and analysed in the range of 400–4,000 cm^−1^.

### 2.4 *In vitro* release behavior of PTMC/DH films

PTMC/DH films of 11 mm in length and 5 mm in width were put in tubes with 5 mL of deionized water. The tubes were then maintained under a temperature shaker at 37°C for 24 h. Then, 5 mL of the eluate was collected and transferred to a centrifuge tube for storage at room temperature, and a further 5 mL of fresh deionized water solution was added to each tube at 2 h, 4 h, 8 h, 12 h, 16 h, 20 h, 1–14 days, respectively. The release amount of doxycycline hydrochloride was measured using a high-performance liquid chromatography (HPLC) (Waters, Milford, MA, United States) with a Waters Model 1515 isocratic HPLC pump to quantify the doxycycline levels in the eluate.

### 2.5 *In vitro* antibacterial activity assay of PTMC/DH films

The activated *Staphylococcus aureus* was transferred to a nutrient solution and incubated at 37°C for 24 h. The bacteria were then appropriately diluted with this nutrient solution. Subsequently, the *Staphylococcus aureus* bacterial solution was removed with a sterile cotton swab and spread evenly over the nutrient agar plates. Three parallel samples were taken at each time point. The solution was then incubated at a constant temperature for 24 h. The diameter of the inhibition ring was measured using a vernier caliper with an accuracy of 0.1 mm, and the area without visible bacterial growth was visually observed as the edge of the inhibition ring. *Staphylococcus aureus* was also incubated with the control pure PTMC films.

### 2.6 Establishment of rat model of Achilles tendon defect

**FIGURE 1:**
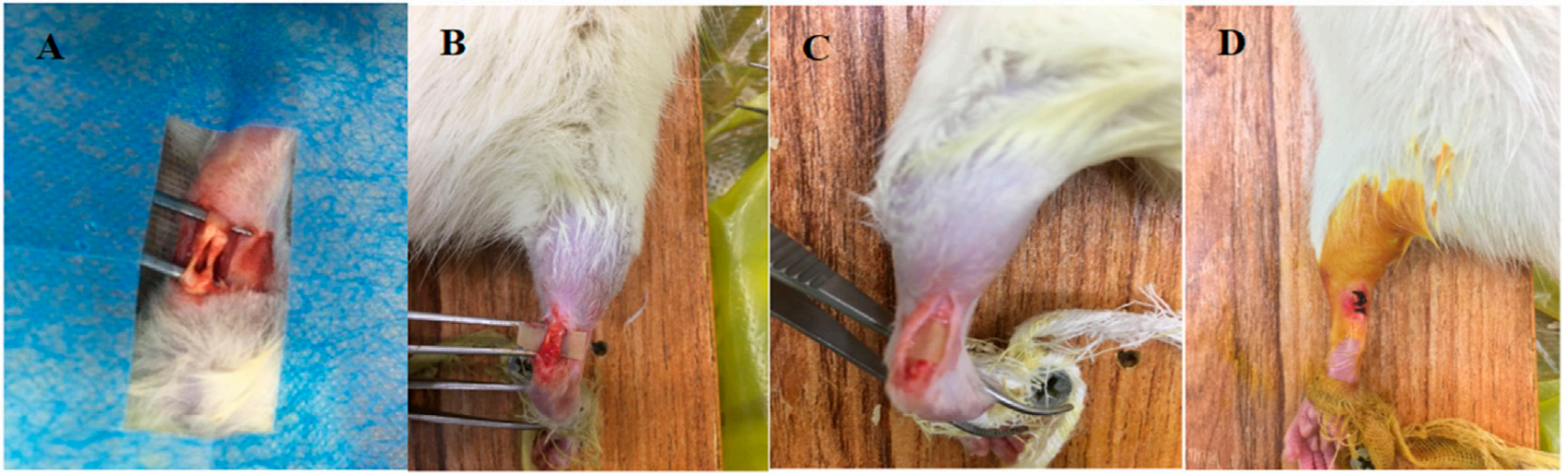
**(A)** Creation of Achilles tendon defect; **(B)** Transfer of PTMC/DH carrier film to Achilles tendon defect; **(C)** Complete encapsulation of Achilles tendon defect with PTMC/DH film; **(D)** Wound Closure.

Twenty-eight SPF male SD rats, 8–10 weeks and 200 ± 50 g in weight, were selected and randomly divided into 7 groups of 4 rats each for *in vivo* Achilles tendon defect repair experiments. The experimental group 1–3 were treated with 10%, 20% and 30% PTMC/DH films, respectively, and experimental group 4 was treated with doxycycline hydrochloride powder covered with pure PTMC films; the control group 5 was treated with pure PTMC films, the blank group 6 was given no treatment, and the healthy group 7 was normal healthy Achilles tendon without any operation. The rats were anesthetized using Pentobarbital sodium, and after sterilization, longitudinal incisions were made on the skin surface of the bilateral (right and left) Achilles tendons of the rats, with a defect of approximately 1/2 of the Achilles tendon, 5 mm longitudinally, as shown below (Figure 1). All operations were performed according to the Regulations of Experimental Animal Administration issued by The people’s Government of Liaoning province.

After surgery, the rat was allowed to move freely in the cage to simulate early functional treatment. two rats of each group were randomly executed at 2 weeks and 4 weeks, respectively. One Achilles tendon specimen was sent for histological analysis and three Achilles tendon specimens were sent for biomechanical analysis in each period.

### 2.7 Analysis of doxycycline levels *in vivo*

Collect 1 mL of blood on each 1, 3, 5, 7, 14, 21, and 28 days, using two centrifuge tubes separated by 0.5 mL each. One copy of the collected blood sample supernatant was sent for pathological analysis and the other for testing by liquid mass spectrometry Liquid Chromatography-Mass Spectrometry (LC-MS). The collected blood samples were centrifuged using a centrifuge and subsequently stored at ultra-low temperature. Centrifugation conditions: centrifuge speed 10,000 rpm for a total of 10 min at −4°C. The amount of doxycycline hydrochloride in the plasma will be determined by LC-MS(Waters H-Class/Xevo TQD, Germany). The mobile phase acetonitrile water (50:50, v/v) was diluted to obtain calibration standard solutions at concentrations of 25, 50, 100, 500 and 1,000 ng/mL^−1^. These standard solutions (10 μL) were diluted with mixed blank plasma (90 μL) to prepare calibration standards containing 5–1,000 ng/mL^−1^ (2.5, 5, 10, 50, 100 ng/mL^−1^). For plasma samples, analytes were extracted from rat plasma using a one-step protein precipitation method in methanol. In a 100 μL rat plasma sample, anhydrous methanol (800 μL) was added to a 1.5 mL centrifuge tube and vortexed for 10 min. The sample was then centrifuged at 14,000 rpm for 10 min at 4°C. The supernatant was then separated from the Eppendorf tube and transferred to a glass culture tube. The sample extract was then dried under a stream of nitrogen at 55°C for 45 min using an MD 200 Sample Concentrator (Liaoning Research Institute of Family Planning, Laboratory Equipment). The residue obtained from this process was then reconstituted in 100 μL of mobile phase and collected in an Eppendorf tube. The solution was then vortex mixed for 50 s, sonicated for 10 min and centrifuged at 14,000 rpm at room temperature for 10 min. The supernatant was transferred to an Agilent LC-MS sample vial and 2 μL was sampled in the LC-MS instrument.

### 2.8 Biomechanical tests

42 rat Achilles tendons collected from 2 to 4 W were moistened with saline, and covered with gauze for testing. The Achilles tendon was fixed on an Instron 5966 universal material testing machine for tensile testing. The Achilles tendon anastomosis was located roughly in the middle of the two tendon clamps. Fixed the Achilles tendon so that the direction of tension was on the same axis as the Achilles tendon, zeroed under complete relaxation of the Achilles tendon, loaded with 0.5 N preload, maintained proper tension on the Achilles tendon and zeroed again. Perform 10 cycles of stretching with a load range of 0–8 N and a loading rate of 3 mm/min. The Achilles tendon was continuously drip-bathed with a soft NS-filled needle during the test. Loaded pull-off test, stretching at a rate of 3 mm/min, with the Achilles tendon not breaking at the jig in the middle position as the criterion for a successful experiment. The computer automatically outputs the maximum load, stiffness and strain at maximum load. After the completion of the mechanical test all specimens are added to a certain amount of Phosphate Buffer Solution (PBS), PH 7.4. They are rapidly frozen with liquid nitrogen and stored for backup.

### 2.9 Pathological histological analysis

A total of 14 root tendons collected from 2 to 4 weeks were subjected to histological examination, fixed in 10% formalin and embedded in paraffin. The embedded tendons were cut into 4 μm cross-sectional sections and stained with hematoxylin and eosin (HE). An independent examiner completed the histological interpretation at magnifications of ×10 and ×20 and calculated the number and density of Fibroblast (Fb) displacements. In addition, a total of 49 serum samples from 7 blood sampling sites were subjected to Enzyme-linked Immunosorbent Assay (ELISA) to test for the pro-inflammatory factor-rat interleukin IL-1β and the anti-inflammatory factor-growth transforming factor TGF-β1 by double antibody sandwich ELISA, both kits were purchased from Rexin Bio (Size 96T, sensitivity: <0.1 pg/mL, IL-1β test range: 1.25–40 pg/mL, TGF-β1 test range: 2.5–80 ng/mL). Operating procedure All reagents and components are first brought back to room temperature, standards, controls and samples, and it is recommended to do duplicate wells. The operating procedure is as follows:

1) Prepare the working solutions for the various components of the kit as described in the previous instructions. 2) Remove the required slides from the aluminium foil bag and put the remaining slides back into the refrigerator in a sealed self-sealing bag. 3) Set up the standard wells, 0-value wells, blank wells and sample wells. Add 50 μL of standard at different concentrations to each of the standard wells, 50 μL of sample diluent to the 0-value wells, and 50 μL of the sample to be tested to the blank wells. 4) Add 100 μL of Horseradish Peroxidase (HRP)-labelled detection antibody to the standard wells, 0-value wells and sample wells, except for the blank wells. 5) Cover the reaction plate with sealing film. Incubate the plate for 60 min at 37°C in a water bath or thermostat protected from light. 6) Remove the sealing film, discard the liquid, pat dry on blotting paper, fill each well with washing solution, leave for 20 s, shake off the washing solution, pat dry on blotting paper and repeat 5 times. If using an automatic plate washer, follow the operating procedures of the washer and add a 30 s soak procedure to improve the accuracy of the assay. At the end of the wash, before adding the substrate, pat the reaction plate dry on clean, non-flaking paper. 7) Mix substrate A and B in a 1:1 volume and add 100 μL of substrate mixture to all wells. Cover the plate with sealing film and incubate for 15 min at 37°C in a water bath or thermostat protected from light. 8) Add 50 μL of termination solution to all wells and read the Optical Density (OD) of each well on an enzyme marker.

## 3 Results

### 3.1 Characterization of PTMC/DH films

**FIGURE 2:**
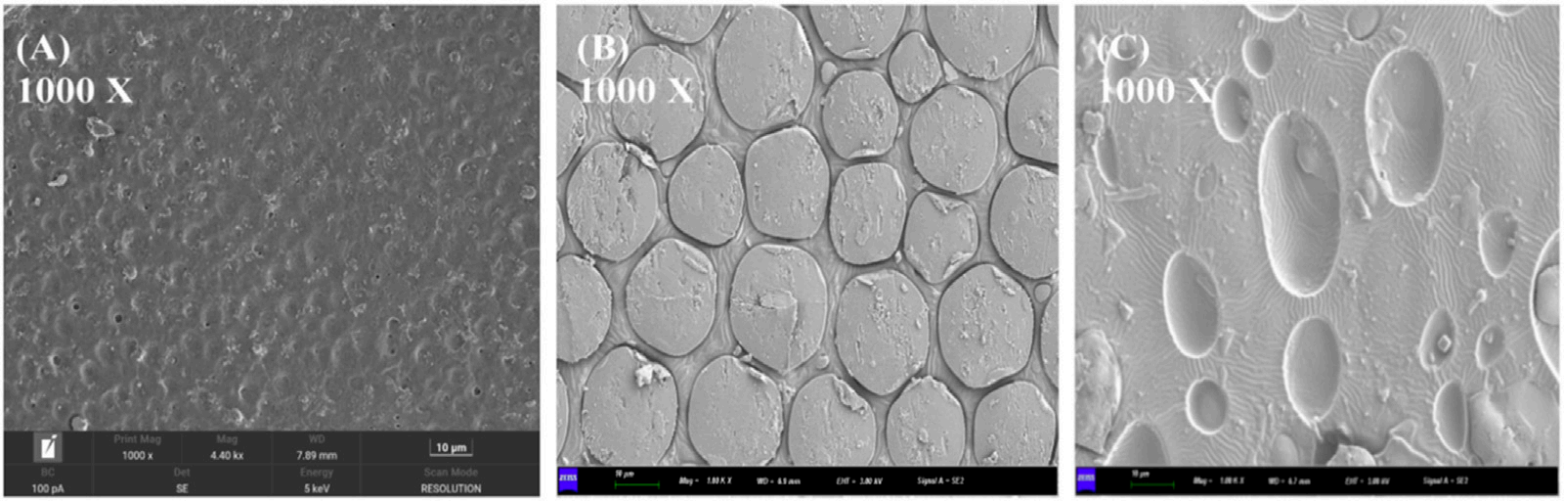
Scanning electron microscope images and magnification distribution of **(A)** 10% drug-loaded film; **(B)** 20% drug-loaded film; **(C)** 30% drug-loaded film.

PTMC/DH films were prepared by the solvent-casting technique. Figures 2A–C shows the SEM images of the drug-loaded films with different concentrations of doxycycline hydrochloride embedded in PTMC. The surface of the PTMC films containing doxycycline hydrochloride is rough. With the amount of doxycycline hydrochloride increased, the grooves and drug particles on the surface of the loaded film become more pronounced.

**FIGURE 3:**
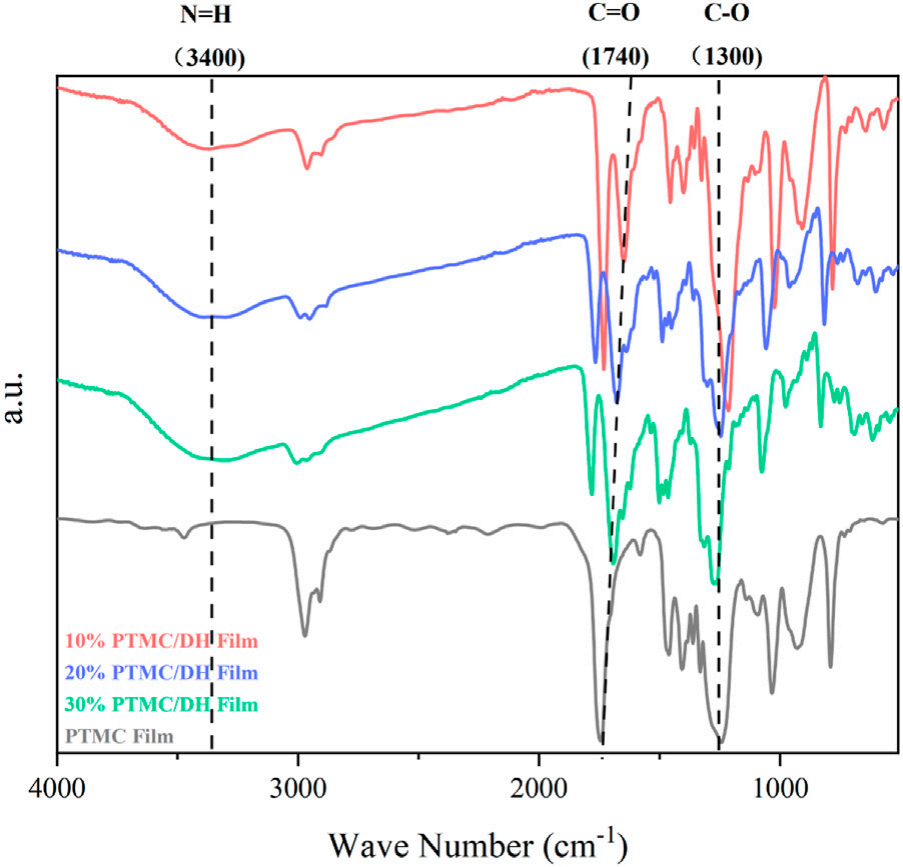
FTIR spectra of PMTC/DH films and PTMC films.

Figure 3 shows the Fourier Transform infrared (FTIR) spectra of the pure PTMC film and the PTMC/DH films. Compared to the spectrum of the PTMC, a new vibrational peak appears at 3,400 cm^−1^ in that of the PTMC/DH films, which can be attributed to the N-H bond of doxycycline hydrochloride, and the peak at around 1,300 cm^−1^ may be the result of increased C-O bonding in doxorubicin (Pasternak et al., 2006; Sun et al., 2017). Furthermore, the vibration at 1,740 cm^−1^ (C=O bond) increases as the DH amount increases in PTMC films. The results of FTIR confimed the successful incorporation of the doxorubicin into the PTMC films. The chemical structure of PTMC has been photographed by using FTIR spectroscopy. Stretching vibrations of the -CH_2_- bond (2,971 and 2,909 cm^−1^) and the C=O bond (1,700 cm^−1^) were observed in PTMC (Han et al., 2010).

To ensure the PTMC/DH films can be degraded *in vivo*, the degradation behavior of PTMC was performed in lipase solutions. There was a significant change in the weight loss (Dry weight) of the pure PTMC films after enzymatic degradation. The results showed that the weight loss was 15.8% ± 1.68%, 35.56% ± 3.69%, 89.90% ± 1.48% at day 1, 3, 9, respectively, indicating an efficient degradation behavior of the pure PTMC under enzymatic digestion, however, the PH values did not change much (Table 1).

**TABLE 1:** Weight loss and PH value of PTMC films after enzymatic digestion.

Time (d)	Weight loss (dry weight)/%	PH
1	15.8 ± 1.68	7.14 ± 0.13
3	35.56 ± 3.69	7.45 ± 0.11
5	49.80 ± 1.88	7.31 ± 0.03
7	64.70 ± 2.21	7.37 ± 0.03
9	89.90 ± 1.48	7.30 ± 0.05

### 3.2 *In vitro* release behavior of PTMC/DH films

**FIGURE 4:**
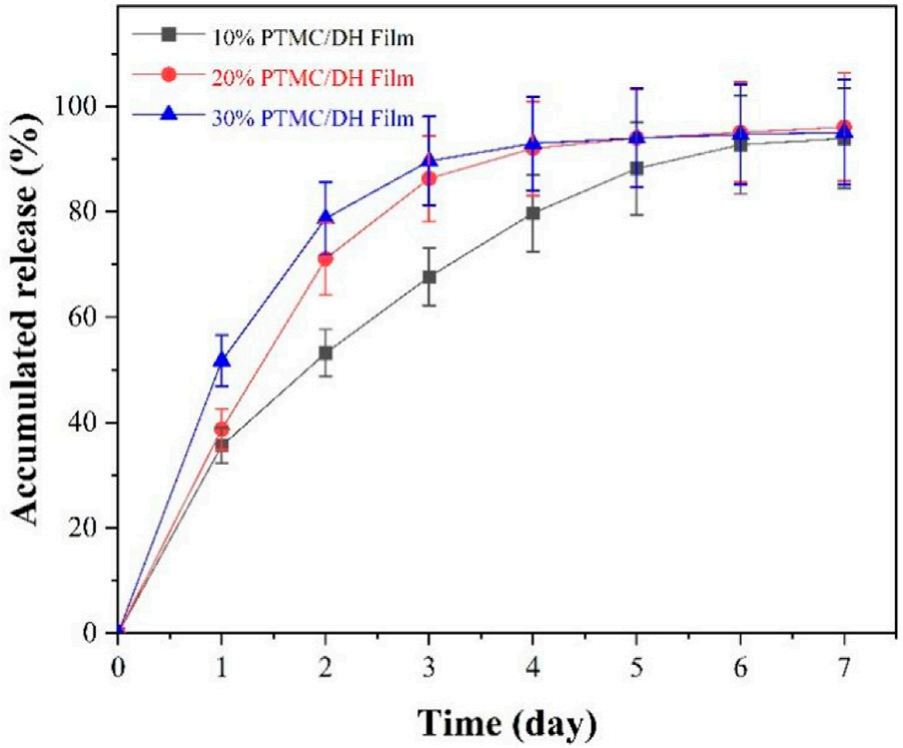
*In vitro* cumulative release of doxycycline hydrochloride.

Figure 4 shows the accumulated release profiles of doxycycline from the PTMC films *in vitro*. The cumulative release profiles suggested that 35.60%, 38.70% and 51.73% of the doxycycline was releasedon day 1, and 67.67%, 86.28%, 89.64% was eluted on day 3 for 10%, 20% and 30% PTMC/DH films, respectively. At 7 days, the accumulated released of doxycycline from all the group reached over 95%. Overall, the PTMC/DH films could provide sustained drug release for more than 7 days *in vitro*, which can be helpful for drug therapy in the acute phase of repairing Achilles tendon defects.

**FIGURE 5:**
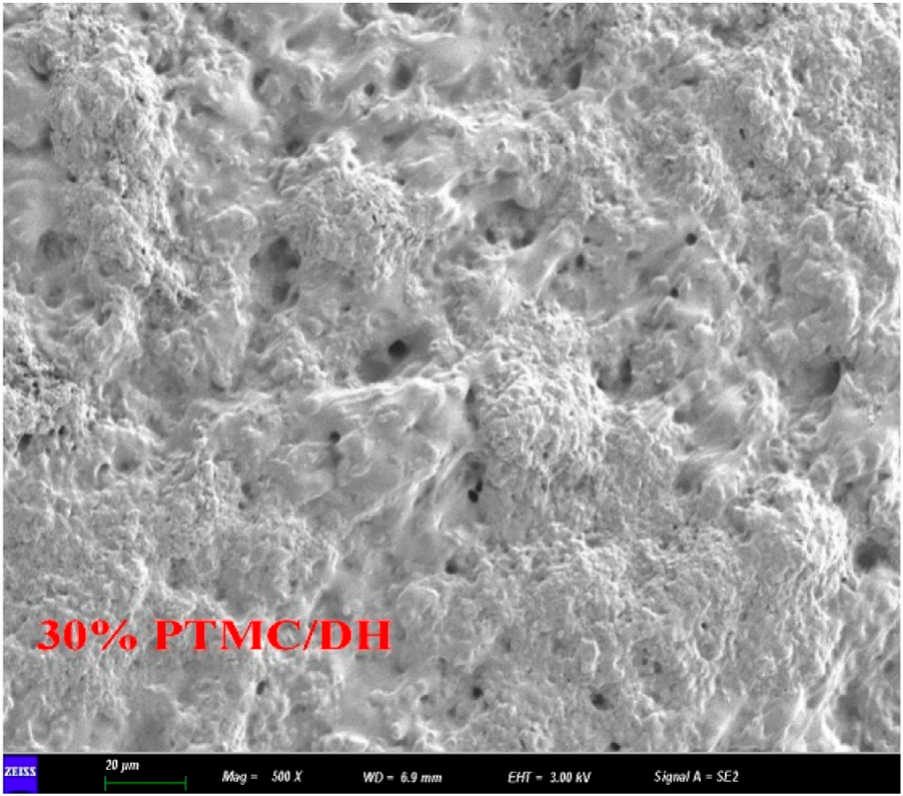
SEM images after hydrolysis of drug-loaded films at different ratios.

After release, SEM images of the remaining drug-loaded films showed that the surface drugs were all diluted, and there were many grooves and pores on the film surface (Figure 5), allowing nutrients to pass through.

### 3.3 The *in vitro* antibacterial effect

**FIGURE 6:**
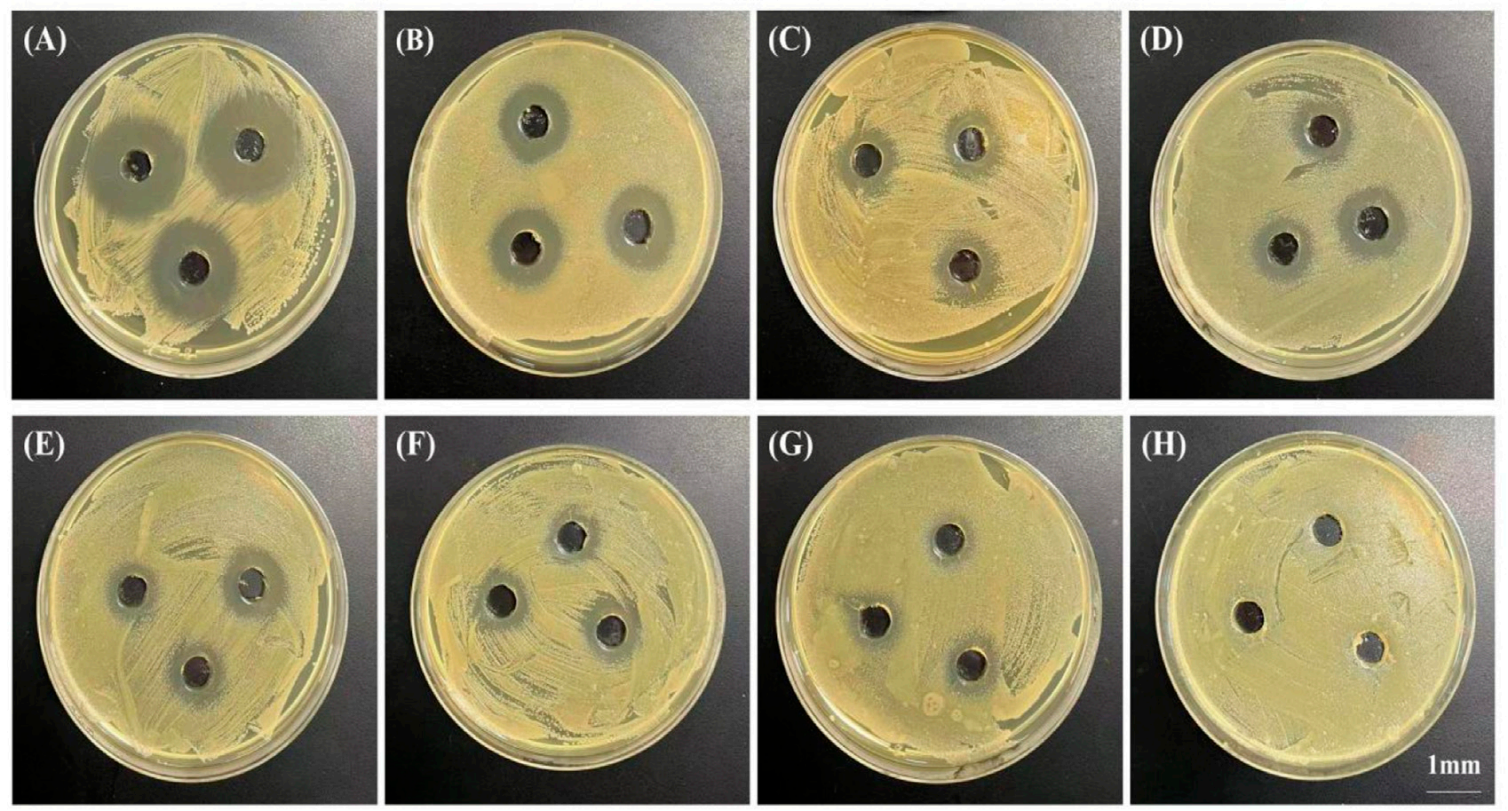
Bacteriostatic rings produced by PTMC-loaded doxycycline hydrochloride loaded drug-loaded films releasing solutions at 2 h **(A)**, 4 h **(B)**, 8 h **(C)**, 12 h **(D)**, 20 h **(E)**; Drug-loaded films at 1 day **(F)** and 3 days **(G)** and by blank PTMC leachate at 1 day **(H)**. The scale bar is 1 mm.

The *in vitro* antibacterial effect was evaluated semi-quantitatively by measuring the diameter of the antibacterial ring (Figure 6). The results showed that at 2 h post-release, the diameter of the antibacterial ring produced by the release solution of 10% PTMC-DH film against *Staphylococcus aureus* was 25.00 ± 1.00 mm; the diameter of the antibacterial ring produced by the release solution of 20% PTMC-DH film against *Staphylococcus aureus* was 29.33 ± 1.15 mm; and that of the antibacterial ring produced by the release solution of 30% PTMC-DH film against *staphylococcus aureus* was 34.67 ± 1.53 mm, showing that as the drug content increased, the diameter of the inhibition ring was also larger, the inhibition effect of its drug-loaded film was more obvious. On the first day of release, the diameters of the inhibition rings of the three ratios were 12.33 ± 2.08 mm, 17.33 ± 1.53 mm and 18.34 ± 1.15 mm, and on the third day of release, the diameters of the inhibition rings of the three ratios were 11.67 ± 0.58 mm, 15.33 ± 1.15 mm and 16.67 ± 1.15 mm. On the fifth day of release, the inhibition rings of the three ratios were 10.67 ± 0.58 mm, 13.67 ± 1.53 mm and 15.33 ± 0.57 mm. In contrast, no inhibition rings were observed in the control group of pure PTMC.

### 3.4 *In vivo* release behavior of PTMC/DH films

**FIGURE 7:**
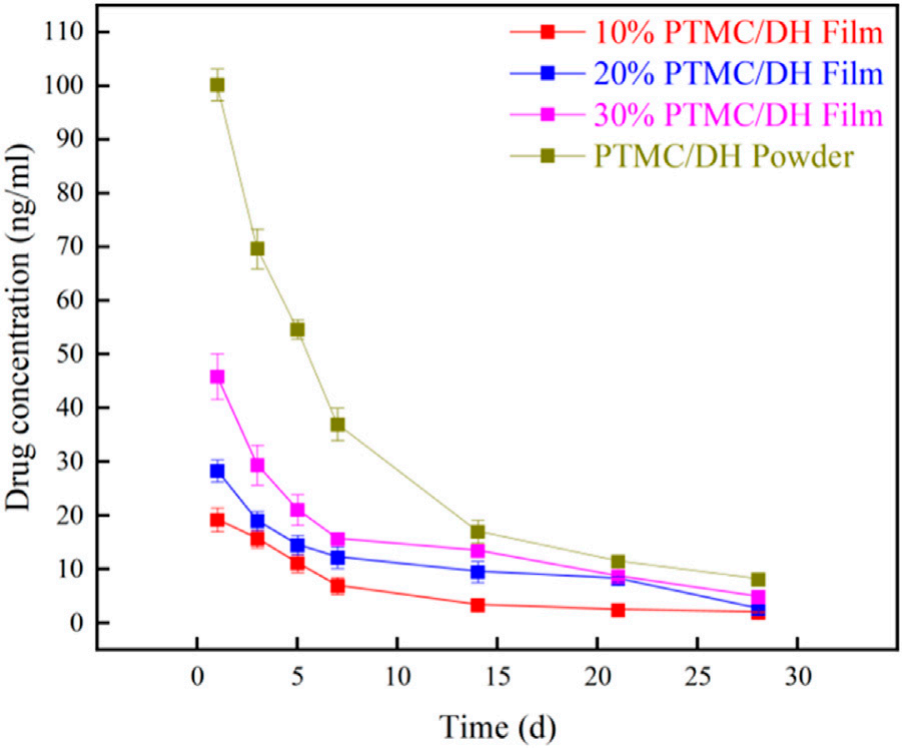
*In vivo* release profile of doxycycline hydrochloride in plasma.

Figure 7 shows the *in vivo* release patterns of doxycycline *in vivo*. A burst release was observed at day 1. After that, the doxycycline level decreased gradually with time, and the drug can sustained release at the tendon for 28 days.

### 3.5 Biomechanical studies

**FIGURE 8:**
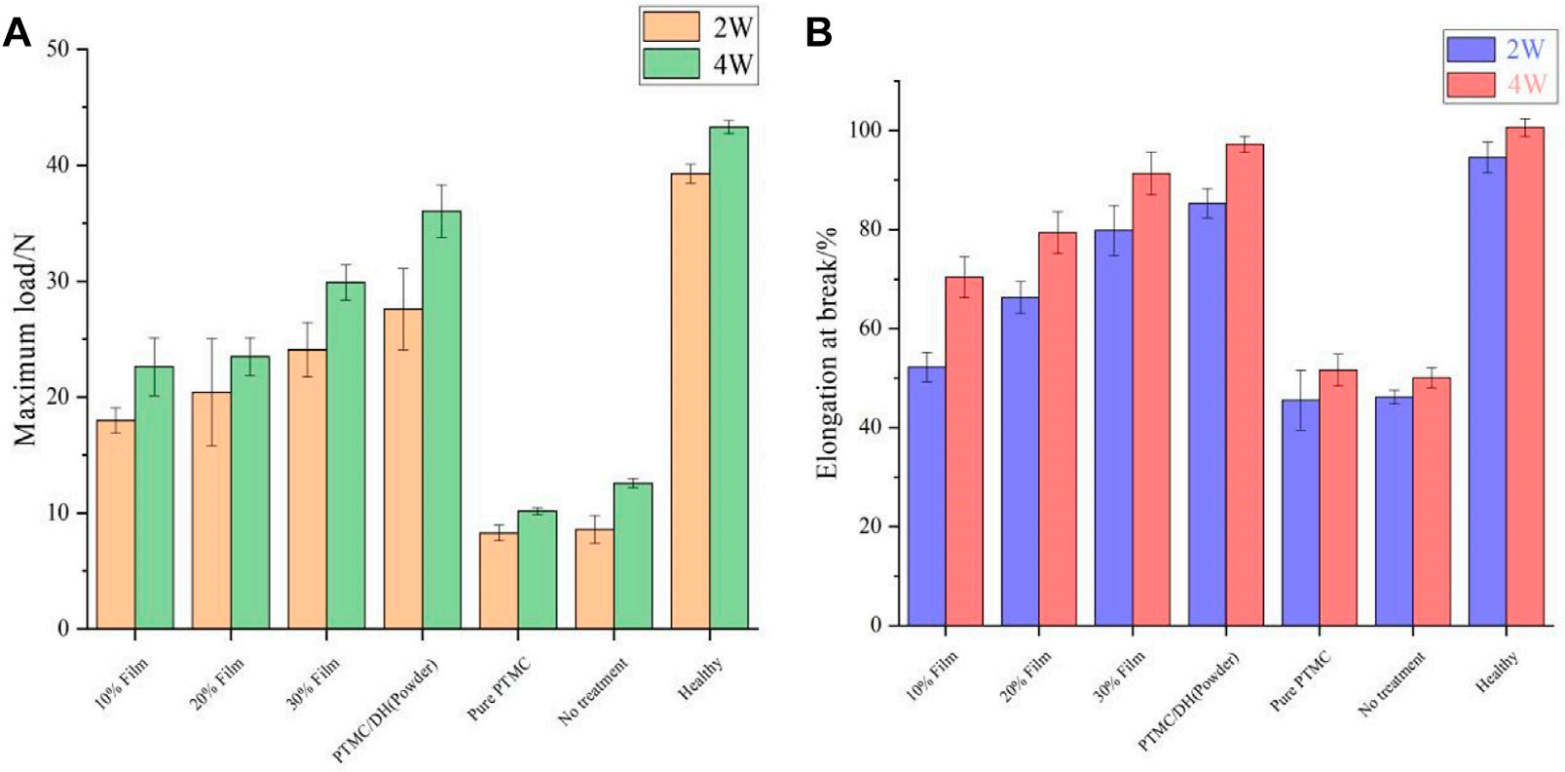
Maximum load **(A)** and elongation at rupture **(B)** of Achilles tendon at 2 and 4 weeks after surgery.

Rats were euthanized 2 and 4 weeks after surgery. The repaired Achilles tendons on both sides of the rats were severed from the proximal calf tendon junction, samples were removed and placed in 10% formalin tissue solution for fixation and biomechanical testing was performed. The maximum load on the repaired Achilles tendon at 2 weeks was gradually increased as the drug content increased from 10% to 30% (Figure 8A); At 4 weeks, the experimental group treated with 30% PTMC/DH films showed much stonger maximum load capacity on the Achilles tendon, which was comparable to the strength level of the healthy group. The control and blank groups without treatment of doxycycline hydrochloride showed much lower maximum strength.

At 2 weeks, the rupture elongation of the repaired Achilles tendon in the experimental was also gradually increased as the DH content increased in PTMC films (Figure 8B); At 4 weeks, the experimental group exhibited a rupture elongation almost close to the level of strength of the Achilles tendon in the healthy group, which also proved that the rats’ Achilles tendon had a greater rupture elongation after the treatment of drug retardation, indicating that the Achilles tendon defect has been well repaired.

**FIGURE 9:**
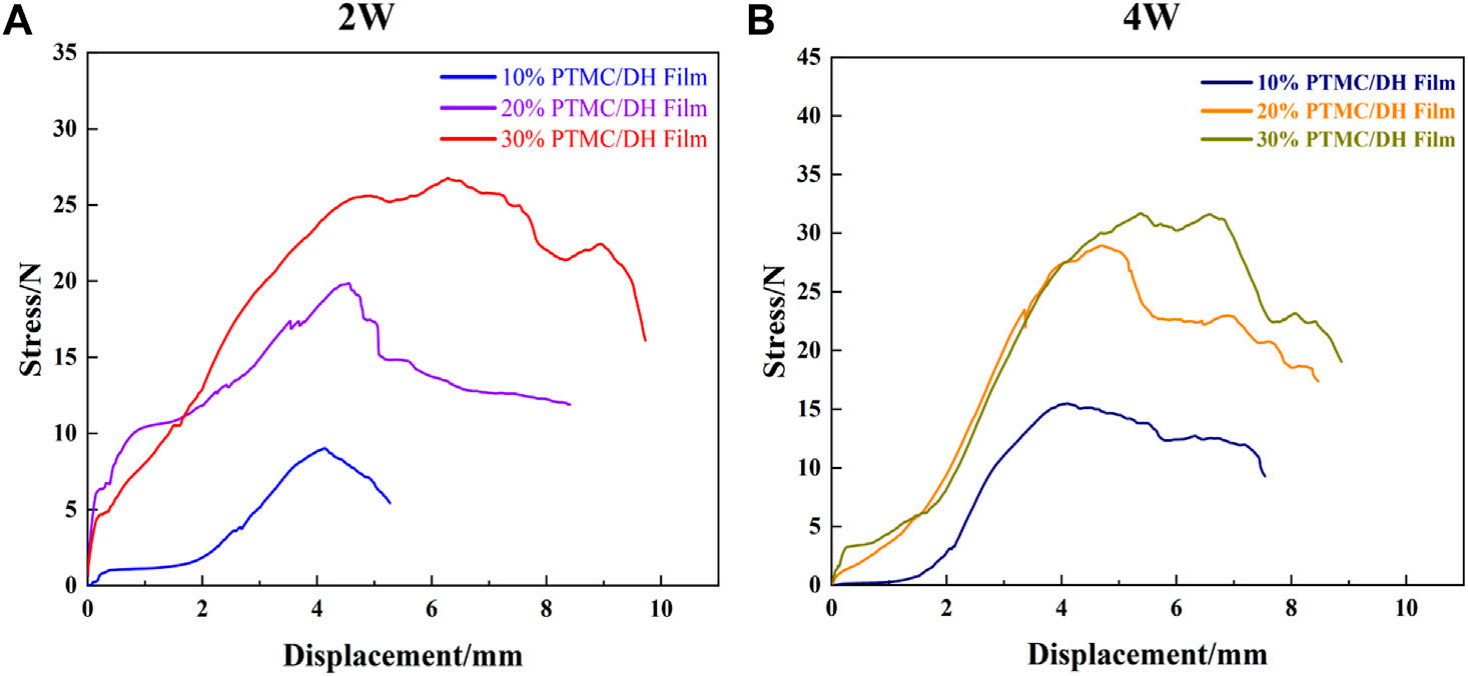
Stress-strain curves of the Achilles tendon after stretching at **(A)** 2 W and **(B)** 4 W for 10%, 20% and 30% drug-loaded films.

In addition, the stress-strain curves of the rat Achilles tendon samples after stretching were also tested and the results showed that the peak of the force-deformation curve tested was significantly greater in the 30% PTMC/DH Film group at 2 weeks than in the 10% and 20% loaded films groups, and the same was true at 4 weeks, but the peak of the overall stress-strain curve at 4 weeks was much improved compared to 2 weeks and the difference was statistically significant (Figure 9). As the area of the stress-strain curve increased at 4 weeks, it demonstrated the greater plasticity, impact resistance and toughness of the repaired Achilles tendon, which was able to withstand greater stress and deformation.

### 3.6 Histopathological study

**FIGURE 10:**
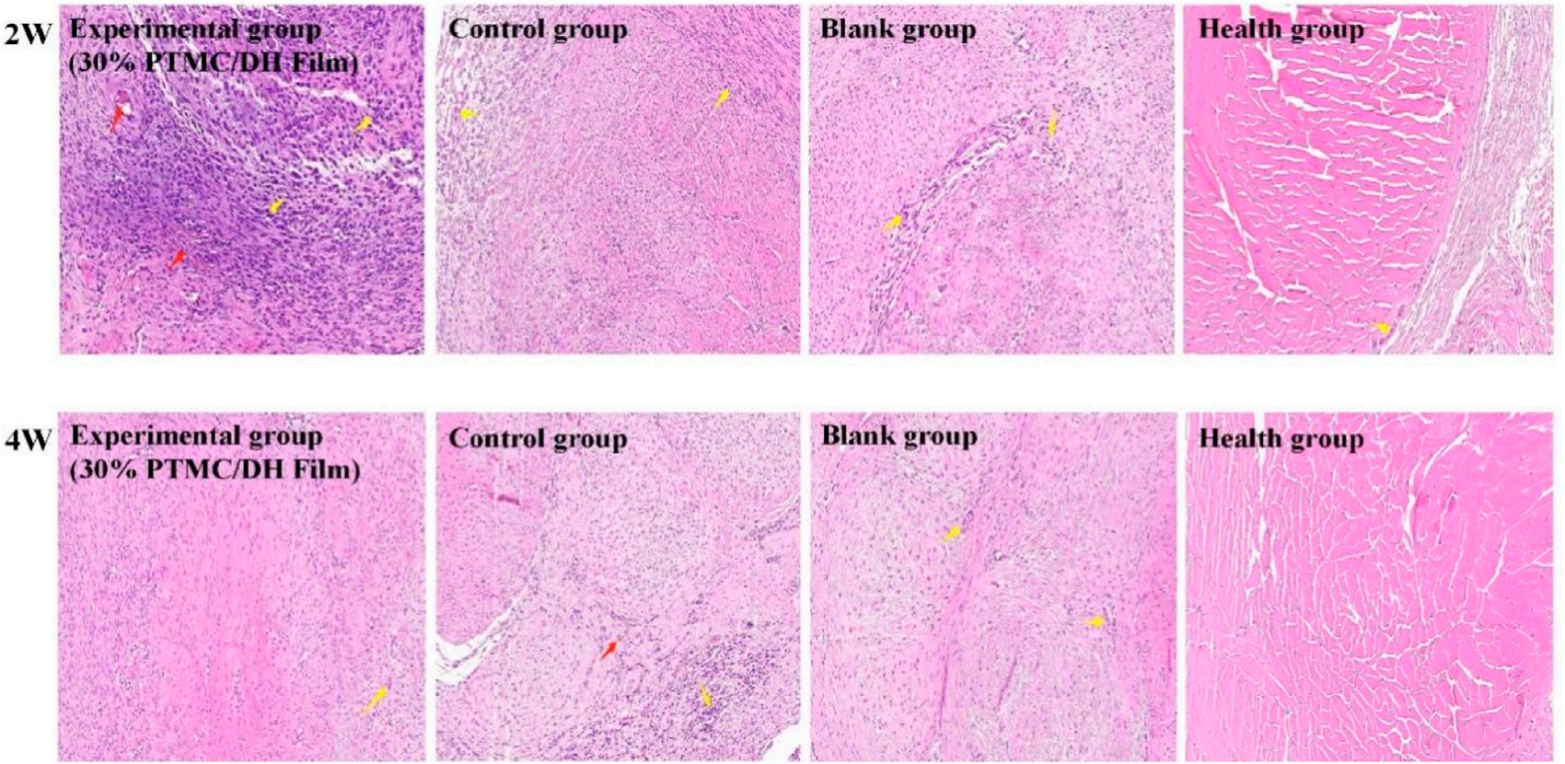
Histology of the rat Achilles tendon at 2 weeks and 4 weeks postoperatively (Red arrows show neovascularization, Yellow arrows show inflammatory cells).

After observing the images obtained after H&E staining for evaluation, it was found that the experimental group did not cause a more severe inflammatory response compared to the control group. However, in the experimental group, at 2 weeks, it was evident that a lot of inflammatory factors and new capillaries could be observed. In contrast, a severe inflammatory response was observed in the control group and in the control group as well as a large collagen fibril component; In the experimental group there was only a slight inflammatory factor at 4 weeks, a few mononuclear inflammatory cells and no neovascularisation, indicating that the Achilles tendon defect had been well repaired (Figure 10). In contrast inflammatory cells were still present in the control and control groups, but were reduced relative to 2 weeks.

**FIGURE 11:**
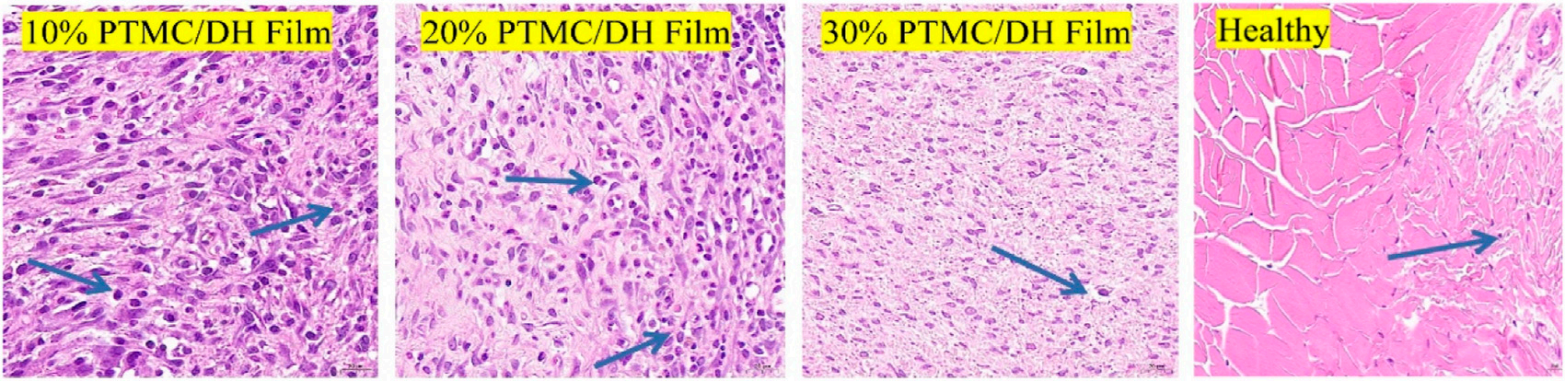
Intercepted field of view images of fibroblast density after 4 weeks of treatment (Blue arrows show fibroblast cells).

**FIGURE 12:**
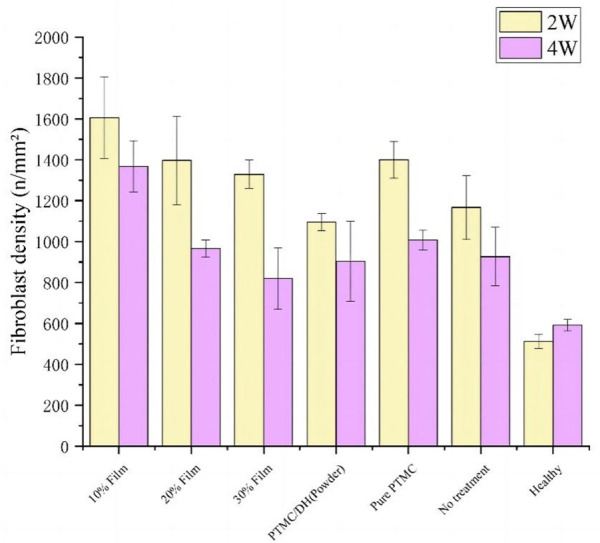
Comparison of fibroblast density value counts between groups at 2 weeks and 4 weeks.

The leading cause of Achilles tendon adhesion after surgery is that the inflammatory reactions at the Achilles tendon injury cause marked hyperplasia of the fibroblast cells, leading to the proliferation of fibrous connective tissue, granulation tissue and scar formation. The target area of tissue was selected for 400× imaging using CaseViewer 2.2 scanning and viewing software, the number of fibroblasts was measured in each section in fields of view using Image-Pro Plus 6.0 analysis software (Figure 11), and fibroblast density was displayed in Figure 12. As seen in Figure 12, the fibroblasts density in the experimental group treated with 10% PTMC/DH films was 1,605.06 ± 199.26 n/mm^2^ at 2 weeks and 1,367.27 ± 125.93 n/mm^2^ at 4 weeks (*p* < 0.01); The density of Fibroblasts in the experimental group treated with 20% PTMC/DH films was 1,396.89 ± 215.95 n/mm^2^, while the density of Fibroblasts in the experimental group treated with 30% PTMC/DH films was 1,329.44 ± 70.67 n/mm^2^ at 2 weeks and 821.44 ± 150.64 n/mm^2^ at 4 weeks (*p* < 0.01); The density of Fibroblasts in the fourth group was 1,095.16 ± 43.11 n/mm^2^ at 2 weeks and 821.44 ± 150.64 n/mm^2^ at 4 weeks (*p* < 0.01); In the control group, the fibroblast density was 1,399.70 ± 89.29 n/mm^2^ at 2 weeks and 1,007.53 ± 47.49 n/mm^2^ at 4 weeks; in the blank group, the Fibroblast density was 1,167.32 ± 156.35 n/mm^2^ at 2 weeks and 926.81 ± 142.77 n/mm^2^ at 4 weeks; The Fibroblast density in the healthy group was 511.67 ± 33.80 n/mm^2^ at 2 weeks and 591.70 ± 28.58 n/mm^2^ at 4 weeks, with no statistically significant difference (*p* > 0.05). The decrease in the density of the fibroblast of the Achilles tendon defect verified the reduction of the inflammatory response of the lesion site after treatment, reducing the phenomenon of Achilles tendon adhesion and helping to achieve sound therapeutic effects.

**FIGURE 13:**
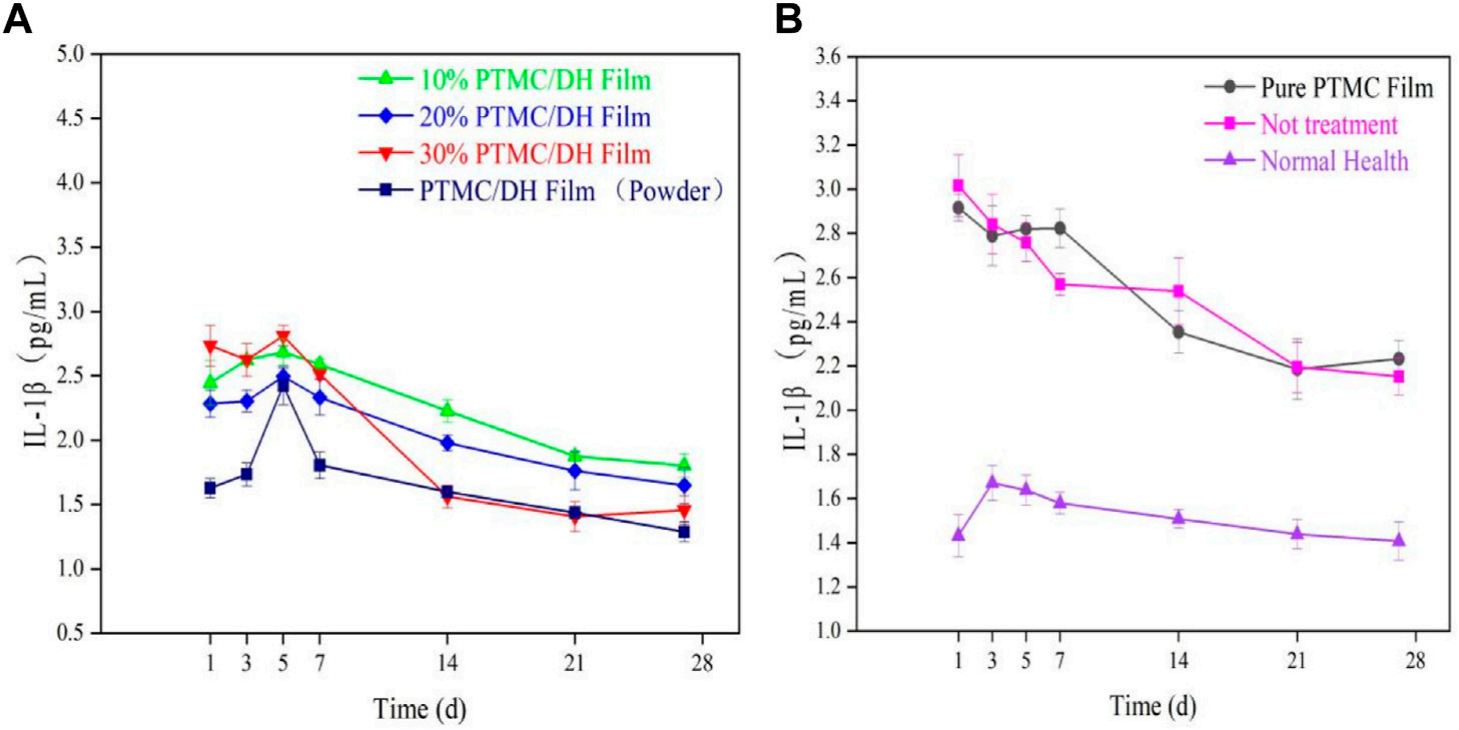
**(A)** IL-1β trend in the experimental group over 28 days; **(B)** IL-1β trend in the control group, blank group and normal healthy group.

After ELISA enzyme-linked immunosorbent assay, the pro-inflammatory factor rat interleukin IL-1β was measured to show a trend of increasing and then decreasing inflammatory response within 28 days (Figures 13A, B). The experimental group showed the highest inflammatory response on 5 days, with a concentration of 2.7558 pg/mL in the 10% drug-loaded film group; 2.4511 pg/mL in the 20% drug-loaded film group; 2.8695 pg/mL in the 30% drug-loaded film group; and 2.3195 pg/mL in the PTMC/DH (Powder) group.

**FIGURE 14:**
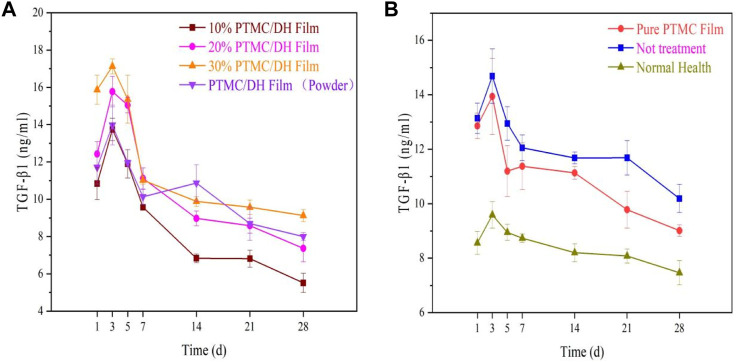
**(A)** TGF-β1 trend in the experimental group over 28 days; **(B)** TGF-β1 trend in the control group, blank group and normal healthy group.

Similarly, the anti-inflammatory factor TGF-β1 was also measured to show an upward and then downward trend, which in turn maintained a dynamic balance with the pro-inflammatory factors in the organism (Figures 14A, B). The highest levels of inflammation were observed in the experimental group at 3 days, with 14.1669 ng/mL in the 10% drug-loaded film group, 15.1968 ng/mL in the 20% drug-loaded film group, 17.4135 ng/mL in the 30% drug-loaded film group and 13.2215 ng/mL in the PTMC/DH (powder) group. Levels were not as pronounced as in the experimental group, which may be related to the addition of the drug, which was able to inhibit the expression of inflammatory factors due to the release of the drug, thus reducing the level of inflammation in the peritendinous area.

## 4 Discussion

As a new era, human standard of living has been further improved. But with this comes problems that have gradually come to the surface. Nowadays sports injuries happen to everyone and Achilles tendon defects in particular have become a popular sports trauma condition. In adolescence, a large amount of physical inactivity leads to a weakening of the muscles around the Achilles tendon, a decrease in soft tissue flexibility and a decrease in the strength of the ligaments to resist external forces. If high intensity sports training is performed, this can lead to damage to the Achilles tendon, which can be severe enough to cause an Achilles tendon defect. Complications following an Achilles tendon defect are not uncommon, with tendon re-rupture being the most common. Postoperative Achilles tendon re-rupture rate of about 7% (Ingvar et al., 2005). Achilles tendon defects are a common injury in sports and other strenuous activities and can lead to functional impairment and disability. The most common clinical approach to human tendon defects is autograft, however this approach leads to donor area morbidity and further debilitation (Jayasree et al., 2019). From a clinical perspective, there is a desire to develop allogeneic materials to complete the repair of Achilles tendon injuries. Biomedical polymers can be used to treat and repair soft tissues, ligaments, muscles and organs that have been damaged by living organisms.

Biodegradable aliphatic polycarbonate has good biodegradability, excellent biocompatibility and physical and mechanical properties (Zhu et al., 1991; Pego et al., 2003; Zhang et al., 2006), and is an important class of biodegradable medical polymer materials. Compared with polyhydroxy carboxylic acid esters and their copolymers (Athanasiou et al., 1996; Karp et al., 2003; Sachlos and Czernuszka, 2003), the greatest advantage of biodegradable aliphatic polycarbonates is that no acidic degradation products are generated during the degradation process (Albertsson and Eklund, 1995), which does not cause side effects such as sterile inflammation in the organism. New photothermally responsive elastomeric composites of aliphatic polycarbonates have been reported, which have efficient self-healing properties and controlled mechanical properties (Chen et al., 2022). The most common and widely studied biodegradable aliphatic polycarbonate as a biomedical material is polytrimethylene carbonate because of its better biocompatibility and degradability (Yang et al., 2014; Yang et al., 2016). MMP is an important bioactive substance *in vivo* and a close relationship between MMP and the degree of recovery of the Achilles tendon has been studied (Garofalo et al., 2011; Mitsui et al., 2012). In the course of past studies, it has been demonstrated that doxycycline hydrochloride is effective in the repair of Achilles tendon defects. Doxycycline hydrochloride is considered to be the most effective inhibitor of MMP among the tetracyclines, and many authors have used doxycycline, a tetracycline, in the repair of Achilles tendon injuries with satisfactory results, and this drug has several clinical applications at this stage (Griffin et al., 2011; Dong et al., 2012). Bedi et al. (2010) had a key role in improving the structural healing rate and success of massive tendon sleeve tears by hypothesizing that doxycycline given immediately postoperatively could accelerate healing by inhibiting collagenase activity. In the rat model, MMP-13 activity has returned to lower basal levels, and therefore doxycycline is less sensitive to its inhibition at 4 weeks postoperatively. Nguyen et al. (2017) used oral doxycycline in a rat model of Achilles tendon defect. The results showed that oral doxycycline accelerated matrix remodelling and promoted the rate of recovery of Achilles tendon defect healing. Pasternak et al. (2006) evaluated the effect of doxycycline on Achilles to Achilles tendon healing in a rat Achilles tendon transection model. Compared to our results, force and energy uptake at the time of disruption was significantly lower in mechanical tests at 5, 8 and 14 days compared to control samples. This study shows for the first time the pharmacological effects of MMP inhibitors on tendon repair. This effect is not necessarily detrimental and, at least in theory, MMP has some potentially beneficial applications in tendon tissue, for example, against tendon damage associated with rheumatoid arthritis (Bramono et al., 2004). Weng et al. (2020) used electrostatic spinning to prepare drug-laden nanofibres and investigated their *in vitro* and *in vivo* drug release. The results showed that Polylactide-glycolic acid (PLGA) copolymer nanofibres released effective concentrations of doxycycline for more than 40 days postoperatively, with lower systemic plasma drug concentrations. Rats implanted with doxycycline nanofibres also exhibited greater postoperative activity and stronger tendons. The findings illustrate the great potential of doxycycline loaded nanofibres in the repair of Achilles tendon ruptures.

In this study, PTMC films were used to embed doxycycline hydrochloride in the drug-carrying film to provide local and sustainable drug release to the lesion site for repair of Achilles tendon defects. However, some of the drug located on the surface of the delivery film then initially shows a burst release, which decreases over time and is excessive in a diffusion-driven manner. *In vivo* drug release peaked on the first day, with the PTMC/DH group releasing the most on the first day, which may be related to the better water solubility of the end of the drug, followed by a gradual decrease in each group until stabilisation around the seventh day. All biomechanical tests showed that the mechanical strength and mechanical properties of the experimental group were better than those of the control and blank groups. Histological analysis showed a gradual decrease in fibroblast density in the experimental group and a tendency for the inflammatory response to first increase and then decrease, both of which were in line with the expected experimental results. In conclusion, this study also demonstrated that locally targeted treatment of Achilles tendon defects in rats also achieved good therapeutic results.

## 5 Conclusion

In this study, we prepared biodegradable polymer-bound doxycycline hydrochloride drug delivery films and evaluated the safety and efficacy of different concentration ratios of drug delivery films in the repair of Achilles tendon defects in rats using PTMC as the carrier material. The experimental group of rats with Achilles tendon defects repaired with doxycycline hydrochloride showed superior mechanical properties and high Achilles tendon activity. Fibroblast density in the experimental group was gradually reduced and inflammatory factor levels were gradually reduced to normal levels. In conclusion, doxycycline hydrochloride containing drug-loaded films showed effectiveness and feasibility in the repair of Achilles tendon defects in rats.

## Data Availability

The raw data supporting the conclusion of this article will be made available by the authors, without undue reservation.
